# The Role of Hydrogen Sulfide in the Localization and Expression of p53 and Cell Death in the Nervous Tissue in Traumatic Brain Injury and Axotomy

**DOI:** 10.3390/ijms242115708

**Published:** 2023-10-28

**Authors:** Stanislav Rodkin, Chizaram Nwosu, Margarita Raevskaya, Maxim Khanukaev, Khava Bekova, Inna Vasilieva, Diana Vishnyak, Anastasia Tolmacheva, Elena Efremova, Mitkhat Gasanov, Anton Tyurin

**Affiliations:** 1 Department of Bioengineering, Faculty of Bioengineering and Veterinary Medicine, Don State Technical University, 344000 Rostov-on-Don, Russia; 2 Department of Instrumentation and Biomedical Engineering, Don State Technical University, 344000 Rostov-on-Don, Russia; 3 Department of Nervous Diseases and Neurosurgery, Rostov State Medical University, 344022 Rostov-on-Don, Russia; 4 Department of Polyclinic Therapy, N.V. Sklifosovsky Institute of Clinical Medicine, I.M. Sechenov First Moscow State Medical University, 119435 Moscow, Russia; 5 Department of Internal Diseases, Surgut State University, Lenina, 1, Nephrology Department, Surgut District Clinical Hospital, Energetikov, 24/3, 628400 Surgut, Russia; 6 Department of Faculty Therapy Named after Professor G.D. Zalessky, Novosibirsk State Medical University, Krasny Prospekt, 52, Department of Medical Rehabilitation, Novosibirsk Regional Clinical Hospital of War Veterans No. 3, Demyan the Poor, 71, 630005 Novosibirsk, Russia; 7 Department of Therapy and Occupational Diseases, Ulyanovsk State University, Lev Tolstoy Street 42, 432017 Ulyanovsk, Russia; lena_1953@mail.ru; 8 Internal Medicine Department, Institute of Medical Education, The Yaroslav-the-Wise Novgorod State University, Derzhavina St. 6, 173020 Veliky Novgorod, Russia; 9 Internal Medicine Department, Bashkir State Medical University, 450008 Ufa, Russia

**Keywords:** traumatic brain injury, axotomy, hydrogen sulfide, apoptosis, necrosis, neuron, glial cells, p53

## Abstract

Traumatic brain injury (TBI) is one of the leading causes of disability and death worldwide. It is characterized by various molecular–cellular events, with the main ones being apoptosis and damage to axons. To date, there are no clinically effective neuroprotective drugs. In this study, we examined the role of hydrogen sulfide (H_2_S) in the localization and expression of the key pro-apoptotic protein p53, as well as cell death in the nervous tissue in TBI and axotomy. We used a fast donor (sodium sulphide, Na_2_S) H_2_S and a classic inhibitor (aminooxyacetic acid, AOAA) of cystathionine β-synthase (CBS), which is a key enzyme in H_2_S synthesis. These studies were carried out on three models of neurotrauma in vertebrates and invertebrates. As a result, it was found that Na_2_S exhibits a pronounced neuroprotective effect that reduces the number of TUNEL-positive neurons and glial cells in TBI and apoptotic glia in axotomy. This effect could be realized through the Na_2_S-dependent decrease in the level of p53 in the cells of the nervous tissue of vertebrates and invertebrates, which we observed in our study. We also observed the opposite effect when using AOAA, which indicates the important role of CBS in the regulation of p53 expression and death of neurons and glial cells in TBI and axotomy.

## 1. Introduction

The most common type of neurotrauma is TBI. This neurotrauma is one of the main causes of death and disability worldwide after cardiovascular and oncological diseases. Unfortunately, to date, the therapeutic strategy aimed at improving outcomes after TBI is not effective enough. One of the main reasons for this situation is the lack of clinically effective neuroprotective drugs that can protect neurons and glial cells of the brain in TBI [1,2].

TBI is characterized by an extensive front of molecular–cellular events that determine the fate of damaged neurons and glial cells, triggering either the processes leading to cell death or the mechanisms of neurocellular recovery. It is well known that powerful oxidative stress and apoptosis are among the main causes of secondary damage to the cells of the nervous system of the brain after TBI. Also, axonal damage to neurons occurs in TBI, which is often characterized by their complete rupture (axotomy) or slow degeneration and can also result in complete axonal collapse and subsequent activation of processes leading to neuron death [3].

To date, many proapoptotic proteins are known that form a complex network of cell death signaling pathways. However, among them there are also key proteins that occupy a central position in the regulation of apoptotic signaling, one of which is p53 [4]. This protein is a transcription factor that triggers the transcription of many proapoptotic genes, and can also implement its program by transcription-independent mechanisms, in particular by regulating mitochondrial functions and triggering apoptosis in cells with mitochondrial deficiency. Its level under normal conditions in the cell is usually maintained low due to its proteasome degradation as a result of ubiquitination by the enzyme ubiquitin protein ligase E3 (MDM2). However, in pathological conditions, including traumatic brain injury and axotomy, the level of p53 increases dramatically, which can lead to cell death. In this regard, of particular interest is the search for signaling mechanisms that can effectively regulate p53 expression [5,6,7]. H_2_S may be a potential candidate.

H_2_S belongs to the class of gasotransmitters-signaling gas molecules synthesized endogenously in the body with the help of special enzymes and performing many functions. For example, H_2_S plays an important role in neurotransmission, regulation of vascular tone, angiogenesis, inflammation, and apoptosis. The following enzymes are responsible for H_2_S synthesis in the body: cystathionine β-synthase (CBS), cystathionine γ-lyase (CSE), and 3-mercaptopyruvate sulfurtransferase (MST) together with cysteine aminotransferase (CAT) [8]. The synthesis of H_2_S in the nervous tissue is mainly carried out by CBS, which is the key enzyme of its endogenous production in the body. It has been shown that the H_2_S concentration is a dynamic system that is very sensitive to pathological changes. Typically, in neurotrauma, H_2_S levels tend to decrease, which adversely affects the survival of neurons and glial cells. H_2_S mainly has a neuroprotective effect by reducing oxidative stress, activating antioxidant enzymes, and reducing the level of neuroinflammation and apoptosis [9,10,11]. Of particular interest are the subtle H_2_S-dependent mechanisms of regulation of proapoptotic signaling through direct interaction with target proapoptotic proteins through S-sulfhydration or persulfhydration of cysteine residues, inhibition or activation of various signaling pathways, etc. [12]. To date, it is known that H_2_S can regulate p53 levels. However, its role in this process is ambiguous: some authors point to its neuroprotective effect due to a decrease in the level of p53 [13], while others demonstrate a cytotoxic effect through the activation of p53 and triggering apoptosis [14,15]. The role of H_2_S in the expression and localization of p53 in neurons and glial cells in TBI and axotomy is barely studied, which makes this research topic particularly relevant and promising both from a practical point of view and from a fundamental standpoint.

In our previous studies, we considered in detail the role of all known gasotransmitters in various pathological conditions [10], and also conducted a review study of H_2_S-dependent signaling mechanisms in the implementation of cell death in injuries of the central and peripheral nervous system and associated neurodegenerative and mental diseases [11]. In addition, we previously studied the role of nitric oxide (NO) in the expression and localization of p53 in neurons and glial cells of the ganglia of the dorsal roots of the rat spinal cord during transection of the sciatic nerve. We managed to establish the NO-dependent mechanisms of p53 deposition in the nucleoplasm during axotomy, as well as the key role of iNOS in this process [16]. A natural continuation of these works was this study, in which we studied in detail the H_2_S-dependent mechanisms of p53 level regulation and cell death in TBI and axotomy.

**Figure 1:**
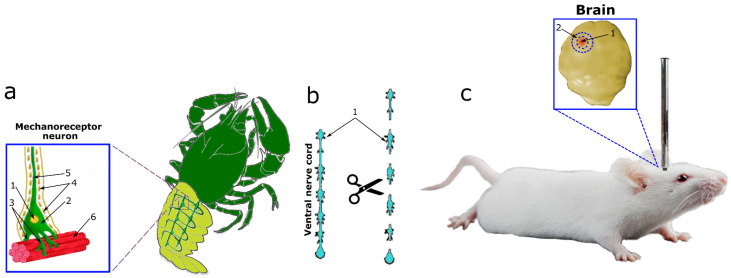
Traumatic brain injury and axotomy models. (**a**) A model of the axotomy of the MRN stretch receptor in crayfish: 1—MRN core; 2—MRN cytoplasm; 3—dendrites; 4—axon; 5—satellite glial cells; 6—receptor muscle. (**b**) Axotomy model of the VNC of crayfish: the VNC consists of 6 ganglia interconnected by connectives, which were cut with ophthalmic scissors. (**c**) A model of traumatic brain injury by dropping the load: 1—necrotic area; 2—area under study, penumbra.

In our research, we studied the effect of a fast H_2_S donor (Na_2_S) and an inhibitor of CBS (AOAA) on the expression and localization of p53 in neurons and glial cells of vertebrates and invertebrates under conditions of neurotrauma. This study entailed the use of two models of neurotrauma (TBI and axotomy) on three experimental subjects of vertebrates and invertebrates (Figure 1). TBI was simulated in mice by dropping a free load on the parietal region of the cerebral cortex according to a standard protocol (Figure 1c). As a model of axotomy, we used the crayfish stretch receptor (CSR) Astacus leptodactylus (Figure 1a) and its axotomized ventral nerve cord (VNC), which were isolated according to the classical method (Figure 1b).

The CSR consists of two mechanoreceptor neurons (MRNs) surrounded by a sheath of satellite glial cells. The axons of the MRN go to the corresponding ganglia of the ventral nerve cord, and the dendrites branch out on the receptor muscles (Figure 1a). MRN axotomy is a classic model for studying axonal stress processes. This model makes it possible to identify neurons and glial cells well by morphological parameters, as well as to study different signaling pathways in the intravital natural neuroglial ensemble. The study of the overlying part of the nervous system, namely VNC ganglia with cut connectives, representing bundles of axons, complements the overall scientific and experimental design of this study well (Figure 1b) [17].

The following methods were used in this study: modeling of TBI on vertebrates, modeling of axotomy on invertebrates, immunofluorescence microscopy, the TUNEL cell death detection method in the mouse brain and morphological analysis of identification of apoptotic bodies in MRN, inhibitory analysis, immunoblotting. These methods made it possible to comprehensively study the role of H_2_S in the expression and localization of p53 in neurons and glia, as well as in cell death in vertebrates and invertebrates during TBI and axotomy. The data obtained will help to better understand the fundamental H_2_S-dependent signaling mechanisms of survival and death of neurons and glial cells in traumatic injury of the nervous system, and the CBS inhibitor and H_2_S donor used in our study can form the basis for the development of clinically effective neuroprotective drugs.

## 2. Results

### 2.1. Immunofluorescent Study of the Role of H_2_S in the Expression and Localization of p53 in Neurons and Glial Cells in Traumatic Brain Injury

**Figure 2:**
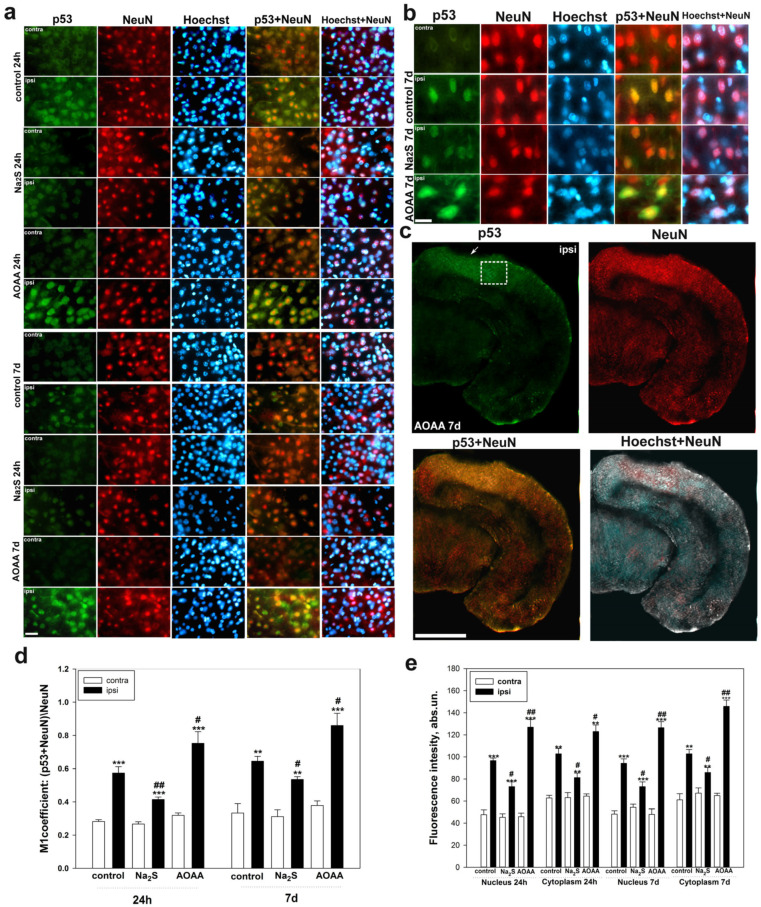
Immunofluorescence microscopy: (**a**) expression of p53 (green fluorescence) in the cerebral cortex of the control group, which was injected with saline, and experimental groups of animals that were injected with Na_2_S and AOAA, 24 h and 7 days after TBI. Scale bar 20 µm. (**b**) Expression of p53 (green fluorescence) in the cerebral cortex of the control group, which was injected with saline, and experimental groups of animals that were injected with Na_2_S and AOAA, 7 days after TBI. Scale bar 25 µm. (**c**) p53 expression (green fluorescence) in a brain section from an animal injected with AOAA, 7 days after TBI. The white square is the area for analysis of p53 expression. White arrow is the location of the injury. Scale bar 500 µm. (**d**) Coefficient M1 of colocalization of p53 and neuronal nuclear marker NeuN in the contralateral and ipsilateral cortex of the control and experimental groups 24 h and 7 days after TBI. (**e**) Dependence of the average intensity of p53 fluorescence in neurons of the contralateral and ipsilateral cortex of the control and experimental groups 24 h and 7 days after TBI. Contra, contralateral cortex; Ipsi, ipsilateral cortex. NeuN, marker of neuron nuclei (red fluorescence); NeuN+p53 and Hoechst+p53, overlap. Hoechst—Hoechst 33342 fluorescence (blue fluorescence), which visualizes the nuclei of all cells, neurons and glia. One-way ANOVA. M ± SEM. n = 6. ** *p* < 0.01, *** *p* < 0.001—ipsilateral cortex relative to contralateral cortex of one animal; # *p* < 0.05, ## *p* < 0.01—ipsilateral cortex of the experimental group versus ipsilateral cortex of the control group within one time period after injury.

**Figure 3:**
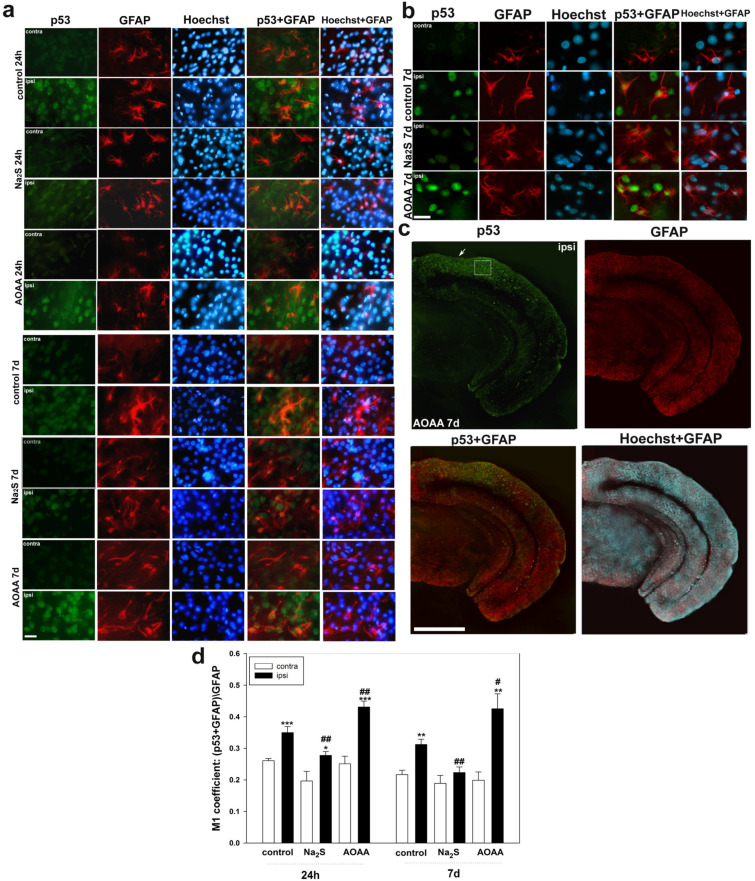
Immunofluorescence microscopy: (**a**) expression of p53 (green fluorescence) in the cerebral cortex of the control group, which was injected with saline, and experimental groups of animals that were injected with Na_2_S and AOAA, 24 h and 7 days after TBI. Scale bar 20 µm. (**b**) Expression of p53 (green fluorescence) in the cerebral cortex of the control group, which was injected with saline, and experimental groups of animals that were injected with Na_2_S and AOAA, 7 days after TBI. Scale bar 25 µm. (**c**) p53 expression (green fluorescence) in a brain section from an animal injected with AOAA, 7 days after TBI. The white square is the area for analysis of p53 expression. White arrow is the location of the injury. Scale bar 500 µm. (**d**) Coefficient M1 of p53 colocalization and GFAP neuronal nuclear marker in the contralateral and ipsilateral cortex of the control and experimental groups 24 h and 7 days after TBI. Scale bar 20 µm. Contra, contralateral cortex; Ipsi, ipsilateral cortex. GFAP+p53 and Hoechst+p53—overlap. Hoechst—Hoechst 33342 (blue fluorescence) fluorescence, which visualizes the nuclei of all cells, neurons and glia. One-way ANOVA. M ± SEM. n = 6. * *p* < 0.05, ** *p* < 0.01, *** *p* < 0.001—ipsilateral cortex relative to contralateral cortex of one animal; # *p* < 0.05, ## *p* < 0.01—ipsilateral cortex of the experimental group versus ipsilateral cortex of the control group within the same time period after injury.

Immunofluorescence microscopy showed that p53 is localized in neurons and glial cells, whose nuclei were detected by Hoechst 33342 fluorochrome (Figure 2 and Figure 3). This is confirmed by the co-localization of p53 with the marker of neuronal nuclei NeuN (Figure 2d) and astrocytes GFAP (Figure 3d). At the same time, the expression of p53 in the ipsilateral and control lateral cortex of the brain of the control and experimental groups, which were injected with H_2_S level modulators, differed significantly. In the contralateral cortex, the level of p53 was insignificant in all groups during all time intervals after TBI modeling. However, traumatic exposure led to a rapid increase in p53 expression in neurons by almost 2 fold in the damaged area of the brain relative to the contralateral area as early as 24 h after injury (one-way ANOVA with Dunnett’s post hoc test, *p* < 0.001, Shapiro–Wilk test *p* = 0.558, Brown–Forsythe test *p* = 0.133). This trend persisted 7 days after TBI (one-way ANOVA with Dunnett’s post hoc test, *p* < 0.01, Shapiro–Wilk test *p* = 0.725, Brown–Forsythe test *p* = 0.204) (Figure 2d).

The use of an H_2_S donor led to a decrease in the p53 level in the ipsilateral cortex relative to the ipsilateral cortex of the control group by 28% (one-way ANOVA with Dunnett’s post hoc test, *p* < 0.01, Shapiro–Wilk test *p* = 0.915, Brown–Forsythe test *p* = 0.192). However, the positive dynamics towards an increase in p53 expression relative to the contralateral cortex of the same animal remained and amounted to 54% (one-way ANOVA with Dunnett’s post hoc test, *p* < 0.001, Shapiro–Wilk test *p* = 0.239, Brown–Forsythe test *p* = 0.769). It should be noted that this significant trend was 2-fold lower than the increase in p53 expression in the control group, which was injected with saline after TBI. Seven days after the injury, the pattern of p53 expression in the nerve cells of the group that received the H_2_S donor was preserved. Thus, the level of p53 increased in the ipsilateral cortex relative to the contralateral cortex of the same animal by 42% (one-way ANOVA with Dunnett’s post hoc test, *p* < 0.01, Shapiro–Wilk test *p* = 0.725, Brown–Forsythe test *p* = 0.204) and decreased relative to ipsilateral cortex of the control group by 18% (one-way ANOVA with Dunnett’s post hoc test, *p* < 0.05, Shapiro–Wilk test *p* = 0.256, Brown–Forsythe test *p* = 0.266) (Figure 2d).

The use of a CBS inhibitor had the opposite effect. Thus, 24 days after TBI, the level of p53 in neurons significantly increased in the ipsilateral cortex of animals injected with AOAA by 34% (one-way ANOVA with Dunnett’s post hoc test, *p* < 0.05, Shapiro–Wilk test *p* = 0.655, Brown–Forsythe test *p* = 0.383) relative to the ipsilateral cortex of the control and 2.2 fold (one-way ANOVA with Dunnett’s post hoc test, *p* < 0.01, Shapiro–Wilk test *p* = 0.072, Brown–Forsythe test *p* = 0.117) relative to the contralateral cortex of the same animal. Similar dynamics persisted 7 days after the injury. Thus, the level of p53 in the ipsilateral cortex of the AOAA group increased by 38% (one-way ANOVA with Dunnett’s post hoc test, *p* < 0.05, Shapiro–Wilk test *p* = 0.471, Brown–Forsythe test *p* = 0.164) and 2.1 fold (one-way ANOVA with Dunnett’s post hoc test, *p* < 0.001, Shapiro–Wilk test *p* = 0.532, Brown–Forsythe test *p* = 0.157) relative to the ipsilateral cortex of the control group and the contralateral cortex of the same animal, respectively (Figure 2d).

The analysis of colocalization of p53 with the nuclear marker NeuN confirms the analysis of fluorescence intensity. Hence, the fluorescence intensity in the nucleus of neurons increased in the ipsilateral cortex of the control group and experimental animals that were injected with Na_2_S and AOAA by 2.1 fold (one-way ANOVA with Dunnett’s post hoc test, *p* < 0.001, Shapiro–Wilk test *p* = 0.948, Brown–Forsythe test *p* = 0.213), by 34% (one-way ANOVA with Dunnett’s post hoc test, *p* < 0.001, Shapiro–Wilk test *p* = 0.406, Brown–Forsythe test *p* = 0.349) and 2.7 fold (one-way ANOVA with Dunnett’s post hoc test, *p* < 0.001, Shapiro–Wilk test *p* = 0.468, Brown–Forsythe test *p* = 0.161) relative to the contralateral cortex 24 h after injury, respectively. The level of p53 decreased in the ipsilateral cortex of Na_2_S-treated animals and increased in the AOAA-treated group relative to the control ipsilateral cortex 24 h after TBI by 31% (one-way ANOVA with Dunnett’s post hoc test, *p* < 0.05, Shapiro–Wilk test *p* = 0.547, Brown–Forsythe test *p* = 0.141) and 30% (one-way ANOVA with Dunnett’s post hoc test, *p* < 0.01, Shapiro–Wilk test *p* = 0.298, Brown–Forsythe test *p* = 0.077), respectively. These dynamics persisted 7 days after TBI (Figure 2e).

Analysis of p53 fluorescence in the neuronal cytoplasm showed similar dynamics, expressed in the following values: the p53 level increased 24 h after TBI in the ipsilateral cortex of the control group and groups that were injected with Na_2_S and AOAA, relative to the contralateral cortex by 64% (one-way ANOVA with Dunnett’s post hoc test, *p* < 0.01, Shapiro–Wilk test *p* = 0.154), Brown–Forsythe test *p* = 0.391), 30% (one-way ANOVA with Dunnett’s post hoc test, *p* < 0.01, Shapiro–Wilk test *p* = 0.777, Brown–Forsythe test *p* = 0.836) and 96% (one-way ANOVA with Dunnett’s post hoc test, *p* < 0.01, Shapiro–Wilk test *p* = 0.226, Brown–Forsythe test *p* = 0.249), respectively. These dynamics persisted 7 days after TBI (Figure 2e).

Next, we examined the level of p53 in astrocytes, which were identified using the specific marker GFAP (Figure 3a–c). Using the analysis of colocalization, we were able to establish that p53 is expressed in this type of glial cells after TBI. In addition, the H_2_S donor and CBS inhibitor had opposite effects on the dynamics of p53 expression in astrocytes after TBI. Consequently, it was shown that the M1 coefficient increased in the ipsilateral cortex of the control and experimental groups, which were injected with Na_2_S and AOAA, relative to the contralateral cortex by 55% (one-way ANOVA with Dunnett’s post hoc test, *p* < 0.001, Shapiro–Wilk test *p* = 0.985, Brown–Forsythe test *p* = 0.098), 41% (one-way ANOVA with Dunnett’s post hoc test, *p* < 0.05, Shapiro–Wilk test *p* = 0.954, Brown–Forsythe test *p* = 0.098) and 73% (one-way ANOVA with Dunnett’s post hoc test, *p* < 0.001, Shapiro–Wilk test *p* = 0.292, Brown–Forsythe test *p* = 0.849), respectively. At the same time, 7 days after injury, there were no significant differences (one-way ANOVA with Dunnett’s post hoc test, *p* = 0.290, Shapiro–Wilk test *p* = 0.313, Brown–Forsythe test *p* = 0.727) in the level of p53 in the ipsilateral cortex relative to contralateral animals injected with an H_2_S donor. Otherwise, the dynamics of p53 expression in astrocytes was preserved 7 days after TBI (Figure 3d).

### 2.2. Western Blot Analysis of the Role of H_2_S in p53 Expression in the Nervous Tissue of the Brain in TBI

**Figure 4:**
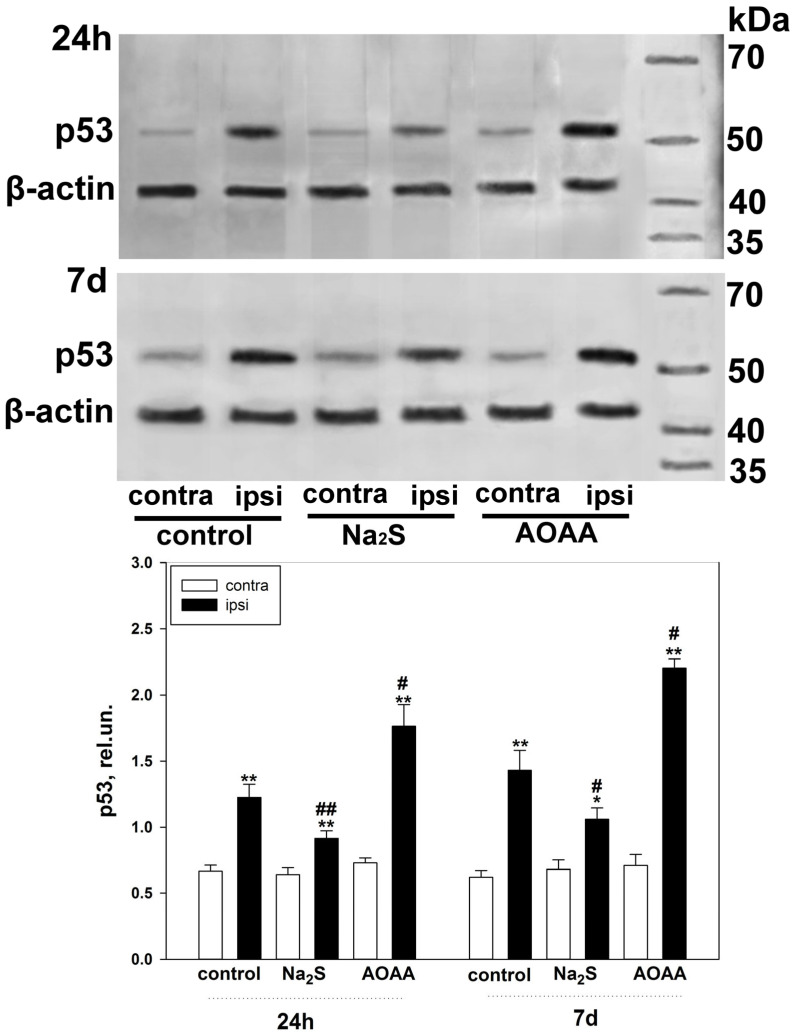
Western blot analysis. Effect of Na_2_S and AOAA on p53 protein expression in the contralateral and ipsilateral cortex 24 h and 7 days after TBI. One-way ANOVA. M ± SEM. n = 6. * *p* < 0.05, ** *p* < 0.01—ipsilateral cortex relative to contralateral cortex of one animal; # *p* < 0.05, ## *p* < 0.01—ipsilateral cortex of the experimental group versus ipsilateral cortex of the control group within one time period after injury.

Fluorescent microscopy data confirm the results obtained during the Western blot analysis of the total fraction of the nervous tissue of the brain of the control and experimental groups, which were injected with Na_2_S and AOAA (Figure 4). Thus, it was seen that p53 expression 24 h after TBI increased in the ipsilateral cortex of the control and experimental groups, which were injected with Na_2_S and AOAA, relative to the contralateral cortex by 71% (one-way ANOVA with Dunnett’s post hoc test, *p* < 0.01, Shapiro–Wilk test *p* = 0.6, Brown–Forsythe test *p* = 0.088), 42% (one-way ANOVA with Dunnett’s post hoc test, *p* < 0.01, Shapiro–Wilk test *p* = 0.057, Brown–Forsythe test *p* = 0.975) and almost 2.5 fold (Kruskal–Wallis H-test, *p* < 0.01, Shapiro–Wilk test *p* < 0.05, Brown–Forsythe test *p* = 0.275), respectively. At the same time, p53 expression in the ipsilateral cortex of animals injected with Na_2_S and AOAA decreased by 36% relative to the control ipsilateral cortex (one-way ANOVA with Dunnett’s post hoc test, *p* < 0.01, Shapiro–Wilk test *p* = 0.245, Brown–Forsythe test *p* = 0.205) and increased by 49% (Kruskal–Wallis H-test, *p* < 0.05, Shapiro–Wilk test *p* < 0.05, Brown–Forsythe test *p* = 0.369), respectively. Similar dynamics persisted 7 days after TBI (Figure 4).

### 2.3. The Role of H_2_S in Cell Death in the Nervous Tissue in TBI

**Figure 5:**
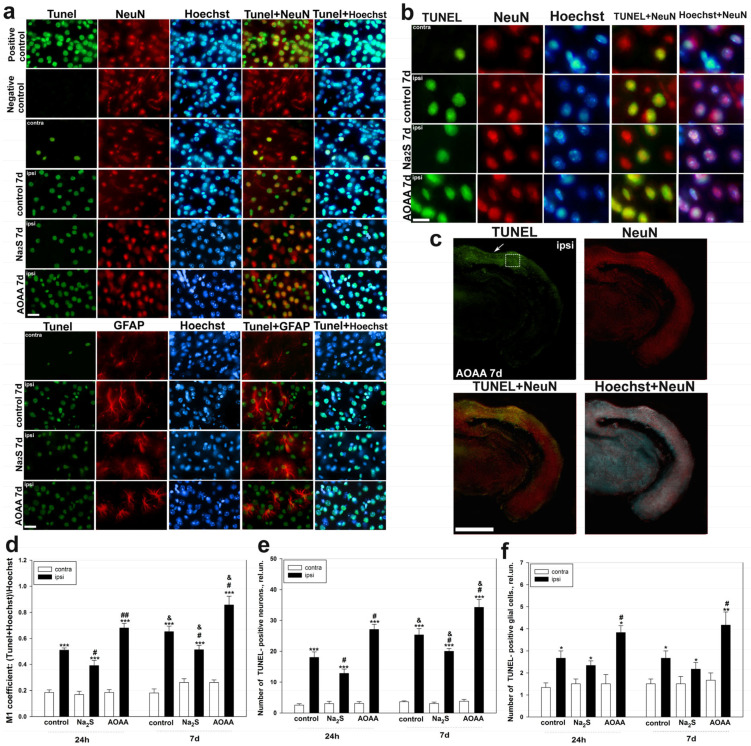
Immunofluorescence microscopy, TUNEL method. (**a**) Cortical sections stained with TUNEL, control group injected with saline, and experimental groups injected with Na_2_S and AOAA, 24 h and 7 days after TBI, as well as sections stained with TUNEL in the presence of nuclease benzonase (positive control) and in the absence of TdT (negative control). Scale bar 20 µm. (**b**) Cortical sections stained with TUNEL, control group injected with saline, and experimental groups injected with Na_2_S and AOAA, 7 days after TBI. Scale bar 25 µm. (**c**) Cortical section stained with TUNEL, experimental group injected with AOAA, 7 days after TBI. The white square is the analysis area for TUNEL-positive cells. White arrow is the location of the injury. Scale bar 500 µm. (**d**) M1 colocalization coefficient of TUNEL and Hoechst cell nuclear marker in the contralateral and ipsilateral cortex of the control and experimental groups 24 h and 7 days after TBI. (**e**) The number of TUNEL-positive neurons in the contralateral and ipsilateral cortex of the control and experimental groups after 24 h and 7 days of TBI. (**f**) The number of TUNEL-positive glial cells in the contralateral and ipsilateral cortex of the control and experimental groups after 24 h and 7 days of TBI. Scale bar 20 µm. Contra, contralateral cortex; Ipsi, ipsilateral cortex. TUNEL, nuclear marker of cell apoptosis (green fluorescence), NeuN, marker of neuronal nuclei or GFAP marker of astrocytes (red fluorescence); TUNEL+NeuN or TUNEL+GFAP and TUNEL+Hoechst—overlap. Hoechst—Hoechst 33342 fluorescence (blue fluorescence), which visualizes the nuclei of all cells, neurons and glia. One-way ANOVA. M ± SEM. n = 6. * *p* < 0.05, ** *p* < 0.01, *** *p* < 0.001—ipsilateral cortex relative to contralateral cortex of one animal; # *p* < 0.05, ## *p* < 0.01—ipsilateral cortex of the experimental group versus ipsilateral cortex of the control group within one time period after injury; and & *p* < 0.05—ipsilateral cortex of the control and experimental groups versus ipsilateral cortex of the same groups at different time intervals after TBI.

Using the TUNEL method, which marks the nuclei of apoptotic cells, we were able to establish that H_2_S plays an important role in the regulation of the death of neurons and glial cells in the brain during TBI (Figure 5a–c). Thus, the colocalization coefficient of M1 TUNEL c Hoechst 33342 showed that 24 h after TBI, there is a pronounced significant trend in the death of nervous tissue cells, which increases with inhibition of the key enzyme of H_2_S synthesis and decreases in the presence of a fast H_2_S donor, and also increases by 7 days. Simultaneously, in the contralateral cortex, which was not subject to traumatic impact, the level of cell death was extremely low in all groups and remained practically at the same level during all time intervals. It was shown that the coefficient M1 ((TUNEL+Hoechst)/Hoechst) increased 24 h after TBI in the ipsilateral cortex of the control and experimental groups, which were injected with Na_2_S and AOAA, relative to the contralateral cortex by 2.7 fold (one-way ANOVA with Dunnett’s post hoc test, *p* < 0.001, Shapiro–Wilk test *p* = 0.304, Brown–Forsythe test *p* = 0.857), 2.3 fold (one-way ANOVA with Dunnett’s post hoc test, *p* < 0.001, Shapiro–Wilk test *p* = 0.162, Brown–Forsythe test *p* = 0.526) and almost 4 fold (Kruskal–Wallis H-test, *p* < 0.001, Shapiro–Wilk test *p* = 0.228, Brown–Forsythe test *p* < 0.05), respectively. At the same time, M1 values in the ipsilateral cortex of animals that were injected with Na_2_S and AOAA decreased by 30% relative to the ipsilateral cortex of the control (one-way ANOVA with Dunnett’s post hoc test, *p* < 0.05, Shapiro–Wilk test *p* = 0.204, Brown–Forsythe test *p* = 0.245) and increased by 33% (Kruskal–Wallis H-test, *p* < 0.01, Shapiro–Wilk test *p* = 0.639, Brown–Forsythe test *p* < 0.05), respectively. Similar dynamics persisted 7 days after TBI (Figure 5d).

We found that the maximum death was observed among neurons after TBI, which increased with the use of AOAA and decreased with Na_2_S. So, 24 h after TBI, in the ipsilateral cortex of the control and experimental groups, which were injected with Na_2_S and AOAA, relative to the contralateral cortex increased by 7.2 fold (one-way ANOVA with Dunnett’s post hoc test, *p* < 0.001, Shapiro–Wilk test *p* = 0.125, Brown–Forsythe test *p* = 0.236), 4.3 fold (one-way ANOVA with Dunnett’s post hoc test, *p* < 0.001, Shapiro–Wilk test *p* = 0.908, Brown–Forsythe test *p* = 0.105) and almost 9 fold (Kruskal–Wallis H-test, *p* < 0.001, Shapiro–Wilk test *p* < 0.05, Brown–Forsythe test *p* = 0.433), respectively. At the same time, the number of neurons in the ipsilateral cortex of animals injected with Na_2_S and AOAA decreased by 38% relative to the ipsilateral cortex of the control (one-way ANOVA with Dunnett’s post hoc test, *p* < 0.05, Shapiro–Wilk test *p* = 0.282, Brown–Forsythe test *p* = 0.929) and increased by 50% (Kruskal–Wallis H-test, *p* < 0.05, Shapiro–Wilk test *p* < 0.05, Brown–Forsythe test *p* = 0.808), respectively. Similar significant trend was observed 7 days after TBI (Figure 5e).

TBI also caused death of astrocytes, but their apoptotic nuclei were found in much smaller numbers. So, 24 h after TBI, in the ipsilateral cortex of the control and experimental groups that were injected with Na_2_S and AOAA, relative to the contralateral cortex increased by 2 fold (one-way ANOVA with Dunnett’s post hoc test, *p* < 0.05, Shapiro–Wilk test *p* = 0.072, Brown–Forsythe test *p* = 0.243), by 60% (one-way ANOVA with Dunnett’s post hoc test, *p* < 0.05, Shapiro–Wilk test *p* < 0.05, Brown–Forsythe test *p* = 0.448) and 2.5 fold (one-way ANOVA with Dunnett’s post hoc test, *p* < 0.05, Shapiro–Wilk test *p* = 0.892, Brown–Forsythe test *p* = 0.304), respectively. At the same time, the number of glial cells in the ipsilateral cortex of animals injected with AOAA 24 h and 7 days after TBI increased by 46% relative to the ipsilateral cortex of the control (one-way ANOVA with Dunnett’s post hoc test, *p* < 0.05, Shapiro–Wilk test *p* = 0.074), Brown–Forsythe test *p* = 0.563) and 61% (one-way ANOVA with Dunnett’s post hoc test, *p* < 0.001, Shapiro–Wilk test *p* = 0.964, Brown–Forsythe test *p* = 0.209), respectively (Figure 5f).

### 2.4. Western Blot Analysis of the Role H_2_S in the Expression of Bax and Bcl-2

**Figure 6:**
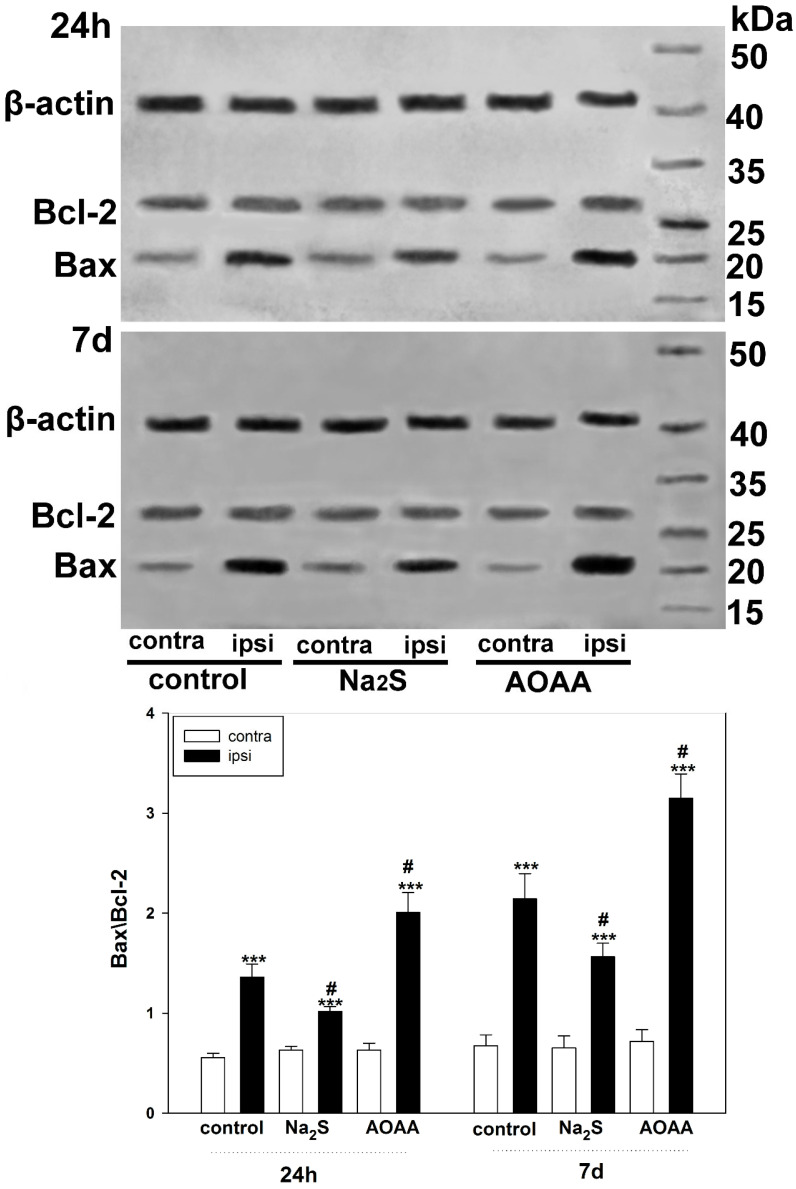
Western blot analysis. Effect of Na_2_S donor and AOAA on the Bax-Bcl-2 ratio in the ipsilateral and contralateral cortex 24 h and 7 days after TBI. One-way ANOVA. M ± SEM. n = 6. *** *p* < 0.001—ipsilateral cortex relative to contralateral cortex of one animal; # *p* < 0.05—ipsilateral cortex of the experimental group versus ipsilateral cortex of the control group within one time period after injury.

Data obtained by the TUNEL method on H_2_S-dependent neuroprotection in TBI were confirmed using Western blot analysis of the ratio of Bax/Bcl-2 proteins, which are key players in apoptosis [18]. Bax is known to stimulate apoptosis by causing permeabilization of mitochondrial membranes. In turn, Bcl-2 blocks the proapoptotic effects of Bax. The balance between these proteins is a critical element in cell survival [19]. We have shown that TBI after 24 h and 7 days causes an increase in the Bax/Bcl-2 index in cells of the damaged cortex relative to the undamaged one by 1.5 fold (one-way ANOVA with Dunnett’s post hoc test, *p* < 0.001, Shapiro–Wilk test *p* = 0.350, Brown–Forsythe test *p* = 0.115) and 2.2 fold (Kruskal–Wallis H-test, *p* < 0.001, Shapiro–Wilk test *p* < 0.05, Brown–Forsythe test *p* = 0.287), respectively. The change in Bax/Bcl-2 in the damaged cortex was due to an increase in Bax levels rather than a decrease in Bcl-2 expression (Figure 6).

This negative effect in the ipsilateral cortex was enhanced when animals were administered an AOAA inhibitor. Thus, the Bax/Bcl-2 index increased in the ipsilateral relative to the contralateral cortex of animals injected with AOAA 24 h and 7 days after TBI by 3.2 fold (Kruskal–Wallis H-test, *p* < 0.001, Shapiro–Wilk test *p* < 0.05, Brown–Forsythe test *p* = 0.338) and 4.5 fold (one-way ANOVA with Dunnett’s post hoc test, *p* < 0.001, Shapiro–Wilk test *p* = 0.996, Brown–Forsythe test *p* = 0.204), respectively. Significant differences were also obtained regarding the ipsilateral cortex of the control group of animals. The Bax/Bcl-2 index increased in the ipsilateral cortex of animals injected with AOAA relative to the ipsilateral cortex of the control group 24 h and 7 days after TBI by 47% (one-way ANOVA with Dunnett’s post hoc test, *p* < 0.05, Shapiro–Wilk test *p* = 0.103, Brown–Forsythe test *p* = 0.740) and 48% (one-way ANOVA with Dunnett’s post hoc test, *p* < 0.05, Shapiro–Wilk test *p* = 0.211, Brown–Forsythe test *p* = 0.794), respectively (Figure 6).

However, the opposite neuroprotective effect was observed when using the H_2_S donor, namely Na_2_S. This effect was expressed in a decrease in proapoptotic activity through a decrease in the level of Bax. Hence, 24 h and 7 days after injury, the Bax/Bcl-2 index decreased in the ipsilateral cortex of animals receiving Na_2_S relative to the ipsilateral cortex of the control group by 17% (one-way ANOVA with Dunnett’s post hoc test, *p* < 0.05, Shapiro–Wilk test *p* = 0.454, Brown–Forsythe test *p* = 0.210) and by 29% (one-way ANOVA with Dunnett’s post hoc test, *p* < 0.05, Shapiro–Wilk test *p* = 0.208, Brown–Forsythe test *p* = 0.496), respectively. Nevertheless, Na_2_S could not completely stop the increase in apoptotic activity in the damaged cortex. At 24 h and 7 days after injury, the Bax/Bcl-2 index increased in the ipsilateral cortex of animals receiving Na_2_S relative to the contralateral cortex of the same group by 40% (one-way ANOVA with Dunnett’s post hoc test, *p* < 0.001, Shapiro–Wilk test *p* = 0.233, Brown–Forsythe test *p* = 0.189) and 1.3 fold (one-way ANOVA with Dunnett’s post hoc test, *p* < 0.001, Shapiro–Wilk test *p* = 0.10, Brown–Forsythe test *p* = 0.786), respectively. It should be noted that this positive trend was significantly lower than in the control group and the group of animals that were administered AOAA (Figure 6).

### 2.5. The Role of H_2_S in the Necrosis and Apoptosis of Neurons and Glial Cells of Crayfish Astacus Leptodactylus during Axotomy

**Figure 7:**
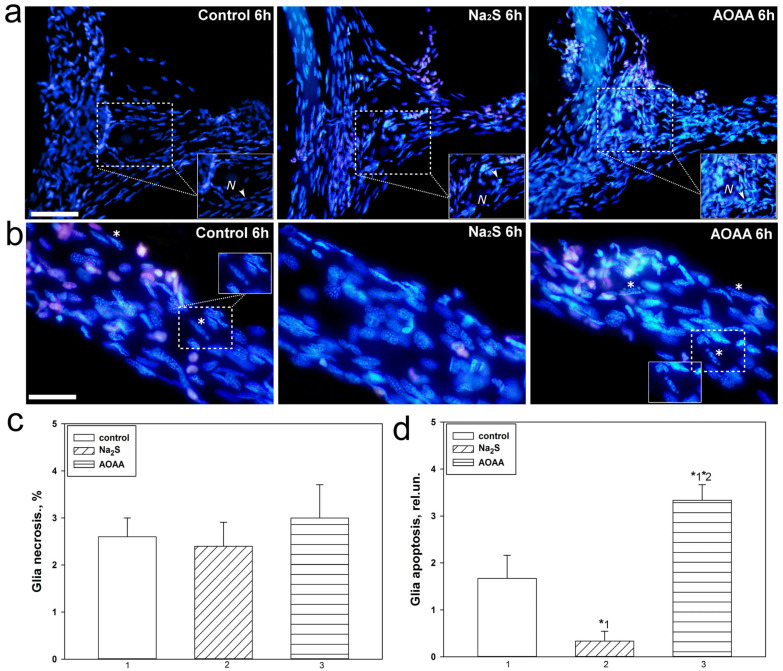
Fluorescence microscopy. (**a**) Crayfish stretch receptor 6 h after axotomy, stained with Hoechst and propidium iodide, control and experimental groups, which were incubated with Na_2_S and AOAA. White dotted square—MRN; N—neuron nucleus; the white arrow—glial cells. (**b**) MRN axon section 6 h after axotomy, stained with Hoechst and propidium iodide of the control and experimental groups. White star—glial cell apoptosis. (**c**) Necrosis of glial cells surrounding the neuron body, 6 h after axotomy. (**d**) Apoptosis of glial cells MRN in a 2 mm area along the axon, was assessed by the number of fragmented glial cells. Scale bar 40 µm. Hoechst+p53—overlap. Hoechst—Hoechst 33342 (blue fluorescence) fluorescence, which visualizes the nuclei of all cells, neurons and glia. One-way ANOVA. M ± SEM. n = 6. * *p* < 0.05.

We studied the role of H_2_S in the necrosis and apoptosis of neurons and glial cells of crayfish Astacus leptodactylus during axotomy. In our study, the necrosis and apoptosis of neurons were not seen, and we barely did detected necrotic nuclei of glial cells stained with propidium iodide (Figure 7a,c). This pattern did not change with the use of Na_2_S and AOAA. However, we observed glial cell apoptosis (Figure 7b), which was significantly reduced by a factor of 5 with H_2_S donor (one-way ANOVA with Dunnett’s post hoc test, *p* < 0.05, Shapiro–Wilk test *p* = 0.392, Brown–Forsythe test *p* = 0.055) and increased almost 2 fold (one-way ANOVA with Dunnett’s post hoc test, *p* < 0.05, Shapiro–Wilk test *p* = 0.497, Brown–Forsythe test *p* = 0.260) when using a CBS inhibitor relative to control drugs 6 h after axotomy. Significant differences were also obtained between groups using Na_2_S and AOAA: the level of glial apoptosis increased in CSR incubated with AOAA, relative to preparations that were incubated with Na_2_S, by 11 fold (Kruskal–Wallis H-test, *p* < 0.05, Shapiro–Wilk test *p* < 0.05, Brown–Forsythe test *p* = 0.243) (Figure 7d).

### 2.6. Immunofluorescence Study of the Role of H_2_S in the Expression and Localization of p53 in Neurons and Glial Cells of Crayfish Astacus Leptodactylus during Axotomy

**Figure 8:**
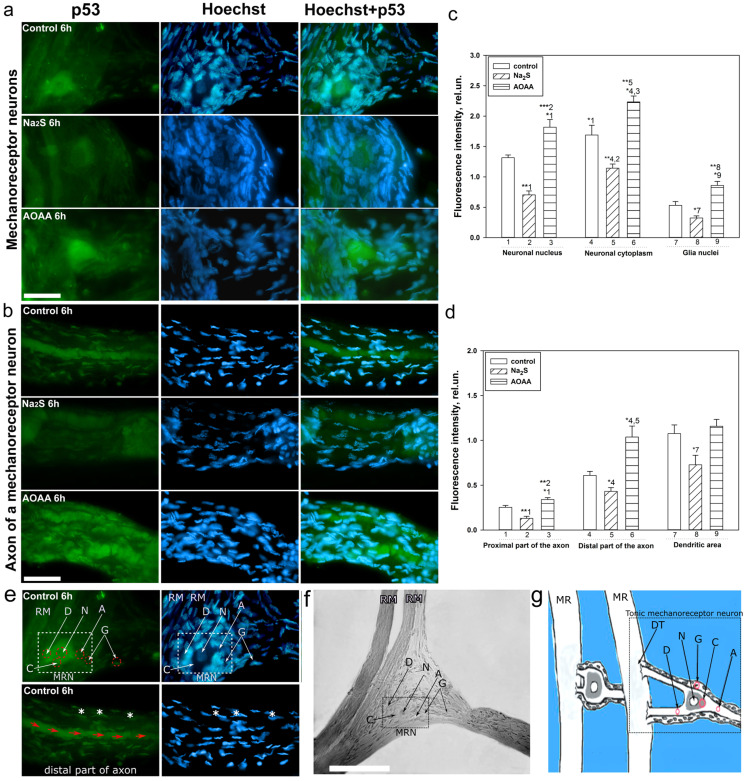
Immunofluorescence microscopy. (**a**) Expression of p53 (green fluorescence) in the stretch receptor of crayfish of the control group and experimental groups that were incubated with Na_2_S and AOAA, 6 after axotomy. (**b**) p53 expression (green fluorescence) in the distal axon region of the MRN of the control group and experimental groups that were incubated with Na_2_S and AOAA, 6 after axotomy. (**c**) p53 fluorescence intensity in the nucleus and cytoplasm of MRN, as well as in the nucleoplasm of glial cells 6 h after axotomy. (**d**) p53 fluorescence intensity in the proximal and distal portion of the axon, as well as in the dendritic region of the MRN. (**e**) Expression of p53 in the stretch receptor of crayfish and distal portion of the axon. (**f**) Crayfish stretch receptor in transmitted light. (**g**) Schematic of the crayfish stretch receptor. RM—receptor muscle; N—neuron nucleus; C—cytoplasm; D—dendrites; A—axon; G and white stars—glial cells; red arrows—p53 cord inside the axon; Hoechst+p53—overlap. Hoechst-Hoechst 33342 fluorescence, which visualizes the nuclei of all cells, neurons and glia. Scale bar 40 µm. One-way ANOVA. M ± SEM. n = 6. * *p* < 0.05, ** *p* < 0.01, *** *p* < 0.01.

Immunofluorescent studies have shown that p53 is localized mainly in the nucleus and cytoplasm of the MRN, as well as in the nuclei of glial cells during axotomy. At the same time, the use of an H_2_S donor and an inhibitor of the key enzyme of its synthesis has opposite effects on the p53 level under conditions of axonal stress (Figure 8a,b). For example, p53 expression in preparations incubated with Na_2_S and AOAA decreased by 48% (one-way ANOVA with Dunnett’s post hoc test, *p* < 0.01, Shapiro–Wilk test *p* = 0.322, Brown–Forsythe test *p* = 0.295) and increased by 31% (one-way ANOVA with Dunnett’s post hoc test, *p* < 0.05, Shapiro–Wilk test *p* = 0.537, Brown–Forsythe test *p* = 0.480) in the cytoplasm relative to controls 6 h after axotomy, respectively. Significant differences in the level of p53 in the cytoplasm were also obtained between the experimental groups (one-way ANOVA with Dunnett’s post hoc test, *p* < 0.001, Shapiro–Wilk test *p* = 0.742, Brown–Forsythe test *p* = 0.596) (Figure 8c).

Expression of p53 in the nucleus was less pronounced than in the cytoplasm (one-way ANOVA with Dunnett’s post hoc test, *p* < 0.05, Shapiro–Wilk test *p* = 0.186, Brown–Forsythe test *p* = 0.165). However, it had a similar dynamic. p53 immunofluorescence in preparations incubated with N_a2_S and AOAA decreased by 87% (one-way ANOVA with Dunnett’s post hoc test, *p* < 0.01, Shapiro–Wilk test *p* = 0.235, Brown–Forsythe test *p* = 0.927) and increased by 38% (one-way ANOVA with Dunnett’s post hoc test, *p* < 0.05, Shapiro–Wilk test *p* = 0.982, Brown–Forsythe test *p* = 0.084) in the nucleus relative to controls at 6 h post-axotomy, respectively. Significant differences in the level of p53 in the nucleoplasm were also obtained between the experimental groups (one-way ANOVA with Dunnett’s post hoc test, *p* < 0.01, Shapiro–Wilk test *p* = 0.966, Brown–Forsythe test *p* = 0.145) (Figure 8c).

In our article, we analyzed the level of p53 in the proximal and distal parts of the axon, as well as in the dendritic region during axotomy. Immunofluorescence of p53 in the proximal part of the MRN axon was weak (Figure 8d). However, significant differences were obtained: the p53 level in preparations incubated with Na_2_S and AOAA decreased by 90% (one-way ANOVA with Dunnett’s post hoc test, *p* < 0.01, Shapiro–Wilk test *p* = 0.513, Brown–Forsythe test *p* = 1) and increased by 36% (one-way ANOVA with Dunnett’s post hoc test, *p* < 0.05, Shapiro–Wilk test *p*= 0.166, Brown–Forsythe test *p* = 0.814) relative to controls, respectively. Significant differences in the level of p53 in the proximal part of the axon were also obtained between experimental groups (one-way ANOVA with Dunnett’s post hoc test, *p* < 0.01, Shapiro–Wilk test *p* = 0.364), Brown–Forsythe test *p* = 0.85). At the same time, we observed a strange accumulation of p53 in the distal part of the axon, which manifested itself in the form of powerful fragmentary strands. We have recorded an increase in the fluorescence intensity of these formations. For example, p53 levels in preparations incubated with Na_2_S and AOAA decreased by 39% (one-way ANOVA with Dunnett’s post hoc test, *p* < 0.05, Shapiro–Wilk test *p* = 0.482, Brown–Forsythe test *p* = 0.858) and increased by 66% (Kruskal–Wallis H-test, *p* < 0.05, Shapiro–Wilk test *p*= 0.991, Brown–Forsythe test *p* < 0.05) 6 h after axotomy relative to control preparations, respectively. In the dendritic region, significant differences were obtained between the control group and samples that were incubated with Na_2_S (one-way ANOVA with Dunnett’s post hoc test, *p* < 0.05, Shapiro–Wilk test *p* = 0.746, Brown–Forsythe test *p* = 0.606) (Figure 8d).

The level of p53 also changed in the nuclei of glial cells (Figure 8a–c). p53 immunofluorescence in preparations incubated with Na_2_S and AOAA decreased by 78% (one-way ANOVA with Dunnett’s post hoc test, *p* < 0.05, Shapiro–Wilk test *p* = 0.775, Brown–Forsythe test *p* = 0.379) and increased by 47% (one-way ANOVA with Dunnett’s post hoc test, *p* < 0.05, Shapiro–Wilk test *p* = 0.771, Brown–Forsythe test *p* = 0.261) in glial cells relative to controls 6 h after axotomy, respectively. Significant differences in p53 levels in glia were also obtained between the experimental groups (one-way ANOVA with Dunnett’s post hoc test, *p* < 0.01, Shapiro–Wilk test *p* = 0.304, Brown–Forsythe test *p* = 0.191) (Figure 8c).

### 2.7. Western Blot Analysis of the Role of Na_2_S and AOAA in p53 Expression in VNC Nervous Tissue during Axotomy

**Figure 9:**
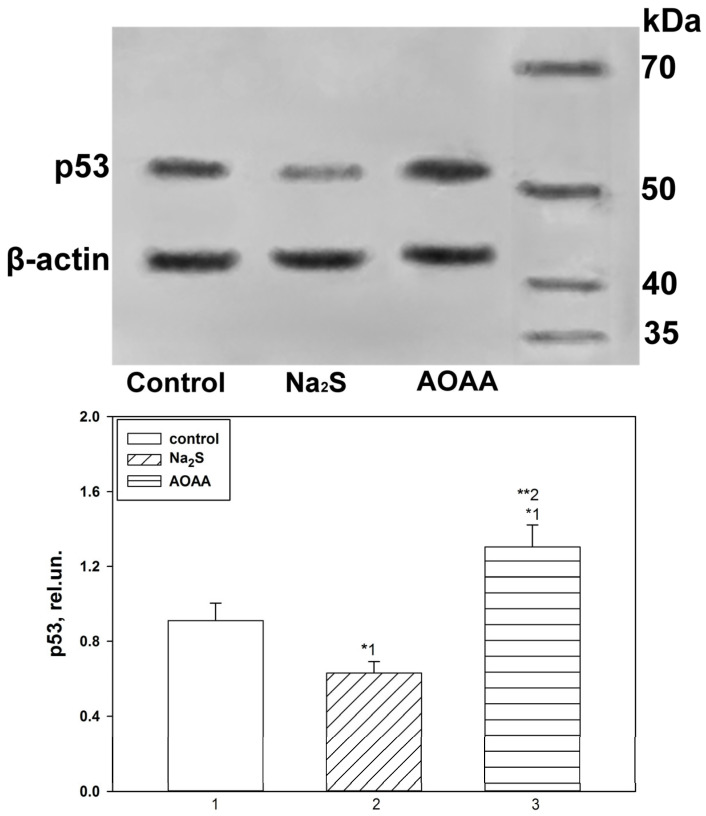
Western blot analysis. Effect of Na_2_S and AOAA on p53 protein expression in axotomized ganglia of the ventral nerve cord 6 h after axotomy. One-way ANOVA. M ± SEM. n = 6. * *p* < 0.05, ** *p* < 0.01.

The fluorescence microscopy data confirm the results obtained during the Western blot analysis of the total fraction of the VNC nervous tissue of the control and experimental groups, which were incubated with Na_2_S and AOAA (Figure 9). Thus, it was shown that p53 expression in samples incubated with Na_2_S and AOAA decreased by 49% (one-way ANOVA with Dunnett’s post hoc test, *p* < 0.05, Shapiro–Wilk test *p* = 0.691, Brown–Forsythe test *p* = 0.510) and increased by 42% (one-way ANOVA with Dunnett’s post hoc test, *p* < 0.05, Shapiro–Wilk test *p* = 0.65, Brown–Forsythe test *p* = 0.546) relative to control 6 h after axotomy, respectively. Significant differences in the level of p53 were also obtained between the experimental groups (one-way ANOVA with Dunnett’s post hoc test, *p* < 0.01, Shapiro–Wilk test *p* = 0.991, Brown–Forsythe test *p* = 0.162) (Figure 9).

## 3. Discussion

Presently, it is known that H_2_S can regulate pro-apoptotic proteins, including p53, in various pathological conditions. The regulation of p53 expression through H_2_S-dependent signaling pathways has been studied in various experimental models using H_2_S donors and inhibitors of the enzymes responsible for the synthesis of this gasotransmitter [13,14,15]. However, the mechanisms of H_2_S-dependent regulation of p53 in neurons and glial cells in traumatic brain injury and axotomy are barely studied.

**Figure 10:**
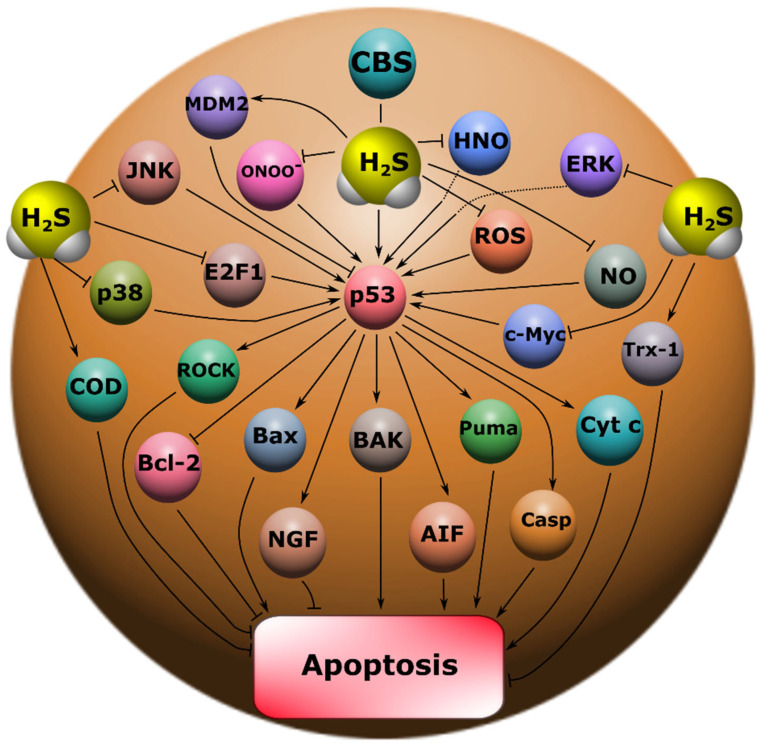
Conceptual scheme of the role of H_2_S in the regulation of p53 levels in TBI and axotomy. Arrows with a sharp end—positive regulation; arrows with a blunt end—negative regulation.

Our study showed that H_2_S plays an important role in the regulation of p53 expression and localization in traumatic brain injury and axotomy (Figure 10). This is consistent with past scientific work reporting H_2_S-mediated effects in modulating p53 levels. However, such studies are few and often contradictory. For example, a recent study demonstrated the neuroprotective effect of H_2_S associated with the inhibition of p53 expression in damaged neurons [13]. At the same time, other authors report that H_2_S induces p53 expression and activates apoptotic signaling [14,15].

The fast H_2_S donor, Na_2_S, and the classical inhibitor of the key enzyme of H_2_S synthesis in the nervous tissue, AOAA, which we used, had opposite effects on p53 expression, both in the TBI model in vertebrates and in the axotomy model in invertebrates. At the same time, the initial level of p53 in intact neurons and glial cells of the brain was at a low level and was slightly higher in the cytoplasmic region than in the nucleoplasm. It is known that p53 expression in normal tissue is tightly controlled by proteasome degradation as a result of ubiquitination by the enzyme ubiquitin protein ligase E3 (MDM2) (Figure 10). Additional control of the p53 level is realized through phosphorylation, acetylation, methylation, glycosylation, as well as the interaction of p53 with other proteins [5,6]. However, cellular stress quickly leads to the activation of p53 through multiple signaling mechanisms [20]. TBI led to a rapid increase in the level of p53 in neurons and glial cells as early as 24 h after injury. These dynamics persisted on the 7th day without a tendency to increase in damaged brain cells. This is probably due to the fact that cellular stress caused by TBI maximally induces transcription of the p53 gene in the first hours after injury [21].

An increase in the intensity of p53 fluorescence both in the nucleus and in the cytoplasm after TBI indicates that p53 can realize its effects through the classical transcription-dependent pathway, also in a non-canonical way. It is known that p53 can trigger apoptotic signaling by binding to mitochondrial Bcl-XL and activating BAK, BAX, and PUMA (Figure 10). This process leads to disruption of the functioning of these intracellular compartments with subsequent permeabilization of their outer membrane, which leads to the release of cytochrome c and AIF, which activate the key proapoptotic protease caspase 3. As a result, cell death occurs via apoptosis [22].

The use of Na_2_S led to a decrease in p53 expression in neurons and glial cells of the damaged cerebral cortex 24 h and 7 days after injury. This effect can be realized through various signaling mechanisms of p53 regulation. Na_2_S is one of the most common fast H_2_S donors in experimental practice, which has shown itself in many studies [23]. H_2_S can reduce oxidative stress and NMDA receptor (NMDAR) activity in damaged cells. It is known that powerful oxidative stress and NMDAR activation are among the main causes of secondary damage to brain cells in TBI [13,24]. H_2_S may protect neurons from oxidative stress by reducing levels of reactive oxygen species (ROS) and lipid peroxidation products. It has been shown that H_2_S can inhibit the biological activity of peroxynitrite (ONOO^−^), which is the most aggressive product of NO metabolism [25]. Also, the antioxidant effects of H_2_S are associated with the reduction of glutathione disulfide (GSSG) and the activation of a number of enzymes of the antioxidant defense system, such as superoxide dismutase (SOD) and thioredoxin (Trx-1) (Figure 10) [9,26]. In turn, it is generally known that ROS induce p53 activation and subsequent apoptotic cell death [27]. NO and its free radical derivatives can also increase p53 expression [28]. In our previous studies, we were able to show that NO increases p53 expression and induces nuclear deposition of this protein in the nucleoplasm in neurons and glial cells under axonal stress [16]. H_2_S can interact directly with NO due to its strong reduction potential to form nitroxide (HNO) [29], which can reduce excitotoxicity through inhibition of NMDAR, thereby exerting a neuroprotective effect (Figure 10) [30]. It should be noted that H_2_S can realize its effects through S-sulfhydration or persulfhydration of cysteine residues on proteins [31].

An opposite effect in increasing the level of p53 in damaged neurons and glial cells 24 h and 7 days after TBI was observed with the use of an AOAA inhibitor that effectively blocks the activity of CBS [32], which is a key enzyme of H_2_S synthesis in the nervous tissue [33]. This result fits well with the general concept of the neuroprotective effect of H_2_S. It should be noted that blocking CBS in many studies led to the development of oxidative stress [34]. The endogenous level of H_2_S and CBS in neurotrauma has a pronounced tendency to decrease up to the minimum values. H_2_S is a dynamic system associated with CBS expression in TBI [35]. Against the background of TBI, additional inhibition of CBS as a result of H_2_S deficiency, will significantly increase proapoptotic signaling.

The study of the role of H_2_S in cell death in the nervous tissue of the brain in TBI was carried out using the TUNEL method. It was shown that TBI leads to apoptosis of neurons and glial cells in the area of damage already 24 h after injury, reaching peak values on the 7th day. Moreover, the death of these cells significantly increased in the ipsilateral cortex relative to the contralateral one, which indicates the important role of apoptotic death in TBI. These results confirm past studies, which have shown that TUNEL-positive response of nerve and glial cells manifests itself already in the first hours after TBI and increases over time, reaching peak values after a few days [36,37]. The use of an H_2_S donor in our study led to a decrease in the level of apoptosis of neurons and glial cells, which confirms the neuroprotective effect of H_2_S. The opposite effect was achieved in groups administered with AOAA. Many studies have shown that CBS plays an important role in the regulation of cell death in neurotrauma [9]. Furthermore, it is reported that the introduction of an exogenous H_2_S donor can restore the CBS level in traumatic brain injury [38]. We suggest that one of the key neuroprotective mechanisms of H_2_S in neurons and glial cells may be a p53-dependent signaling pathway. In our study, the level of p53 expression directly correlated with the administration of various H_2_S modulators under conditions of traumatic injury to the nervous tissue.

We also examined the role of H_2_S in the regulation of Bcl-2 and Bax expression to confirm the activation of apoptotic signaling in TBI. Bcl-2 is known to inhibit apoptosis by blocking the release of mitochondrial apoptogenic factors such as cytochrome c and AIF, and is a molecular antagonist of the proapoptotic protein Bax, which causes permeabilization of mitochondrial membranes [39,40]. The balance between the levels of these proteins is one of the key factors determining the fate of a cell: survival or death. A number of authors have shown that H_2_S can act as a modulator of the level of these proteins, regulating the mitochondrial pathway of apoptosis [41]. Our study demonstrated that H_2_S reduces Bax levels in traumatic brain injury. In this case, inhibition of CBS has a pronounced opposite effect. One of the indirect H_2_S-dependent mechanisms of Bax regulation may be the modulation of L-type Ca^2+^ channels [42]. In addition, H_2_S can activate the PARP1/Bax signaling pathway, damaging brain cells [43]. However, the use of cysteine analogues, which increase H_2_S production, did not affect Bcl-2 levels. Yet, they increased the expression of Bax and p53, which indicates the ambiguity of the role of H_2_S in the regulation of proapoptotic signaling [44].

In the second part of our study, we studied the H_2_S-dependent mechanisms of p53 localization and expression, as well as cell death in MRN and surrounding glial cells and VNC of invertebrate animals using the example of crayfish *Astacus leptodactylus* under conditions of axonal stress caused by complete axon transection. We pursued the goal of studying in detail the expression of p53 in different parts of MRN and VNC, as well as cell death in PPP under the action of Na_2_S and AOAA during axotomy. Previously, we have already studied the expression and localization of p53 in these invertebrate axotomy models [17]. In the current study, we were able to establish that Na_2_S and AOAA have opposite effects on the level of apoptosis of glial cells during axotomy. The H_2_S donor showed a protective effect and reduced glial apoptosis 6 h after injury, while AOAA showed a cytotoxic effect. To date, it is known that CBS is expressed in invertebrates. This enzyme is the most important regulator of many metabolic processes [45] in the nervous system of crustaceans [46] and freshwater crayfish Procambarus clarkii [47].

However, the role of CBS and H_2_S in the localization and expression of p53 and cell death in the nervous tissue of the crayfish *Astacus leptodactylus* and in general in crustaceans has not been studied. The results obtained indicate an important role of H_2_S in the regulation of apoptotic signaling induced by axotomy. It should be noted that we did not observe significant differences in satellite glial cell necrosis during axotomy between control and experimental neuroglial preparations. We also did not observe fragmentation of neuron nuclei, which is characteristic of the terminal stage of apoptosis, as well as necrosis of these cells. Probably, the absence of MRN apoptosis is associated with insufficient time for its development, and MRN necrosis usually does not occur during axotomy [17]. MNR apoptosis has not been observed in other studies under axonal and photodynamic stress, as well as in pharmacological modeling of various signaling pathways [47,48].

A detailed analysis of the localization of p53 in CSR showed that this protein is localized in the nucleus, cytoplasm, axon, and dendrites of the MPH, as well as in the nucleoplasm of satellite glial cells. Na_2_S caused a decrease in the level of p53 fluorescence in different parts of the MPH, and inhibition of CBS by AOAA had the opposite effect. Intensive accumulation in MRN and surrounding glia may indicate the intensification of processes associated with apoptosis induced by axotomy. Moreover, here. we see that H_2_S plays an important role in the implementation of p53-dependent signaling under conditions of acute axonal stress. Probably, H_2_S can influence the mechanisms of nuclear cytoplasmic tralocation of p53. It is widely accepted that p53 is synthesized in the cytoplasm and then transported to the cell nucleus. Unbound p53 forms a complex with MDM2, which rapidly transports it back to the cytoplasm, where it undergoes proteasomal degradation. Its accumulation in the MRN nucleus may be associated with intense synthesis and rapid post-translational translocation into the nucleoplasm. It can also be caused by hampered nuclear-cytoplasmic transport. It is well known that p53 has nuclear localization signals (NLS) and nuclear export signals (NES), which allows it to efficiently move between the nucleus and cytoplasm [49]. These sites can be a potential target for H_2_S interaction with them, which can modify their activity. It should be noted that H_2_S can modulate the activity of various protein kinases [50]. It is known that kinases MAP JNK, p38, ERK, etc. (Figure 10), are important molecular players in the regulation of p53 activity through the mechanisms of phosphorylation of this protein [51]. Since axotomy does not cause DNA damage, potential signals for p53 synthesis include transcription factors such as E2F1, c-Myc, p38, as well as the universal second messenger H_2_S. An increase in the level of these proteins was found in axotomized VNC ganglia [52]. In a recent study, we found an increase in the expression of E2F1 in MRN, VNC of crayfish, as well as ganglia of the dorsal roots of the spinal cord during axotomy [53]. It has been shown that H_2_S can regulate the expression of E2F1 [54].

Interesting results were obtained on the H_2_S-dependent localization and expression of p53 in the proximal and distal parts of the axon, as well as in the dendritic region of the MRN during axotomy. We observed that the level of p53 was barely observed in the proximal part of the axon. However, in the distal part of the axon, an accumulation of p53 in the form of pronounced fragmented strands was found. The p53 fluorescence intensity in this region decreased with the use of the H_2_S donor and increased with CBS inhibition. In recent years, information has appeared on the role of p53 in the regulation of growth and regeneration of axons. Inhibition of p53 blocks NGF-dependent axon growth. In cells exposed to NGF, an increase in p53 level [55] and a decrease in MDM2 expression [56] were found. Importantly, phosphorylated p53 can directly interact with Rho kinase (ROCK) in the axon, which promotes axonal growth by regulating the actin and microtubule system (Figure 10) [55]. Perhaps this is the effect we observed in our study.

Our study reveals new H_2_S-dependent signaling pathways regulating cell death under conditions of mechanical injury. This may help in the development of new clinically effective neuroprotectors and treatment strategies for TBI and nerve damage caused by complete rupture. It is worth noting that these pathological conditions are directly associated with mental disorders [11], which are accompanied by increased oxidative stress and apoptosis [57,58]. Many studies indicate that p53 may play an important role in mental disorders [59,60]. In turn, H_2_S is an important link in the pathogenesis of these diseases [11,61]. In addition, p53 and H_2_S play an important role in neurodegenerative diseases [62,63]. Therefore, our results can be considered in the context of these pathological conditions and are of interest to specialists in this field.

It is known that today there are no clinically effective neuroprotective drugs that can protect neurons and glial cells from traumatic effects. Many scientific works indicate that H_2_S is a critically important signaling molecule with a pronounced cytoprotective effect [9,10,11,62,63]. Today, the most popular and scientifically recognized H_2_S donors are Na_2_S, calcium sulfide (CaS) and NaHS [8]. We have shown that Na_2_S can exert a neuroprotective effect through the regulation of the expression level of p53, as well as Bcl-2 and Bax (Figure 10). Therefore, drugs based on Na_2_S and its modifications can be considered as potential candidates for neuroprotectors.

The cytotoxic effect of AOAA that we observed, manifested in an increase in p53 expression and apoptosis during TBI and axotomy, indicates an important role for CBS in these pathological conditions. Therefore, the development of selective CBS activators is a very promising direction, which may be the key to obtaining an effective drug capable of protecting cells of the nervous system from mechanical damage. Unfortunately, selective CBS activators have not yet been developed. So far, the best known allosteric activator of CBS remains S-adenosyl-L-methionine (SAM) [64].

It is worth noting that the results obtained are another step in understanding the complex mechanisms in the survival and death of neurons and glial cells under conditions of traumatic injury. However, the human brain, with its complex neuromolecular ensemble, poses a serious challenge in fundamental and applied neuroscience, since there are always limitations in extrapolating results obtained from animals to it. That is why it is important to continue research in this area in order to apply it as objectively as possible to clinical practice in humans.

## 4. Materials and Methods

### 4.1. Animals

Experiments on traumatic brain injury were carried out on male CD-1 mice aged 14 to 15 weeks and weighing 20–25 g. Animals were kept in standard cages in groups of 6–7 individuals with free access to food and water. The animals were kept under standard conditions: cycle 12 light/12 dark, 22–25 °C, air exchange rate 18 shifts per hour. International, national and/or institutional guidelines for the care and use of animals have been followed.

Axotomy experiments were carried out on crayfish *Astacus leptodactylus* from the tributaries of the Don river. Animals were kept in special containers filled with water under the same standard conditions.

The studies were carried out in accordance with the requirements of the Directives of the Council of the European Community 86/609/EEC on the use of animals for experimental research (24 November 1986), “Rules of laboratory practice in the Russian Federation” (Order No. 708n dated 23 August 2010), and organization of procedures when working with laboratory animals” (GOST 33215–2014) and Protocol No. 2 approved by the Commission on Bioethics of the Don State Technical University on 17 February 2020.

### 4.2. Object and Procedure

The model of experimental traumatic brain injury was modeled according to the standard protocol, which was further modified. Mice were anesthetized with an intraperitoneal injection of chloral hydrate (300 mg/kg). After anesthesia, the animals were placed in a specially designed system for modeling craniocerebral injury, fixed to accurately determine the location of the traumatic impact. Then, a focal injury of the right hemisphere of the brain with an area of approximately 9 mm^2^ was induced using a weight drop device consisting of a metal rod (with a tip 3 mm in diameter, 5 mm long) weighing 200 g. The weight was dropped from a height of 3 cm onto an unexposed skull. The load drop coordinates were set as 2 mm dorsally from the bregma, 1 mm laterally from the midline (Figure 1c). The area of damage is the parietal region of the cerebral cortex. The weight loss model is one of the most common animal models of TBI [65]. To model TBI in mice, we relied on two protocols that were previously described in articles [66,67]. However, we reduced the weight from 333 g to 200 g, increasing the drop height of the load to 3 cm [66], which reduced the degree of damage to the nervous tissue of the brain and significantly increased the survival rate of mice. We did not observe spontaneous cessation of breathing or seizures in mice after injury. Also based on the second protocol, we decided not to traumatize the mice through surgery, which involved exposing the skull for impact and subsequent suturing [67]. We sought to reproduce as closely as possible the real conditions for the occurrence of TBI. An autopsy showed a small area of necrosis at the site of the blow and moderate swelling in the damaged hemisphere of the brain. After recovery from anesthesia, the mice were returned to their usual home. In this study, two groups of animals were formed: control and experimental.

The following modulators were used to study H_2_S-dependent mechanisms of p53 regulation, as well as to study the effect of H_2_S on the survival and death of neurons and glial cells in TBI: H_2_S donor sodium sulfide (Na_2_S, 0.1 mg/kg; Khimikon, Krasnodarskiy Kray, Russia) [68] or CBS inhibitor aminooxyacetic acid (AOAA, 5 mg/kg; Tianjin Xidian Chemical Technology Co., Ltd., Tianjin, China) [69], which were administered intraperitoneally after TBI and then daily for 7 days until decapitation. The control group of mice was injected with saline. The control group of mice was injected with saline solution. Na_2_S is a low molecular weight compound that can effectively release H_2_S. In many studies, Na_2_S has established itself as a classical H_2_S donor [68,70]. In turn, a classic inhibitor of CBS, the key enzyme of H_2_S synthesis in nervous tissue, is AOAA, which has found wide use in experimental practice [69,71].

All animals for experiments were selected on a blind basis without any assessment of appearance and behavioral characteristics.

Axotomy was performed on two simple experimental neuroglial preparations of crayfish Astacus leptodactylus. The first drug was the CSR, which consists of two MRNs surrounded by satellite glial cells and a pair of receptor muscles. It was isolated according to the classical method of Florey and Florey, as a result of which two neurons axotomize during their isolation (Figure 1a) [17,72]. Mechanoreceptor neurons have several large dendrites that branch on the muscle into smaller branches, and an axon. During muscle stretching, the dendritic membrane is depolarized and a receptor potential is generated. One of the neurons generates potentials only when the receptor muscle is stretched. Another neuron generates potentials constantly, which makes it possible to assess the level of its viability [48,72]. We used the latter type of MRN in our study.

Two CSRs are located in each segment of the abdomen on its inner surface from the dorsal side. MRNs have several large dendrites and an axon. The receptors were isolated with parts of the chitin shell to which they were attached and placed in a special cuvette filled with 2 mL of van Harreveld’s physiological solution for cold-blooded animals (mM: NaCl—205; KCl—5.4; NaHCO_3_—0.24; MgCl_2_—5.4; CaCl_2_—13.5; pH 7.2–7.4). The cuvette was equipped with one fixed hook and another, manipulatory, capable of setting the degree of stretching of the receptor muscles. Pieces of chitinous shell were mounted on these special devices.

MRN viability was assessed by recording action potentials from the axon. This procedure was carried out extracellularly using a glass suction microelectrode filled with van Harreveld’s saline solution. Action potentials were enhanced by a bioelectrical activity amplifier. Changes in the frequency of action potentials were recorded continuously until the end of incubation after axotomy. The cessation of bioelectrical activity was interpreted as the functional death of the neuron. Such MRNs were not used in this study.

The ganglia of VNC crayfish were used as the second preparation. The VNC consists of 6 ganglia connected by connectives, which are powerful bundles of axons that connect ganglion neurons and transmit information to the head ganglia. Ophthalmic scissors were used to cut the connectives between the ganglia (Figure 1b). Then, the axotomized ganglia were placed in a cuvette with van Harreveld’s solution.

The studies were carried out at a temperature of approximately 22–24 °C for 6 h from the moment of axotomy. To study the role of H_2_S in the regulation of expression and localization of p53, as well as cell death of neurons and glial cells in axonal injury, similar modulators were used as in TBI at the following final concentrations: Na_2_S (250 μM) or AOAA (3 mmol). These modulators were added to the cuvette immediately after axotomy. Neuroglial preparations were incubated with them for 6 h.

### 4.3. Immunofluorescence Microscopy

Determination of localization using immunofluorescent analysis of p53 in the brain 24 h and 7 days after TBI was performed according to the following protocol. The area around the focus of necrosis caused by the impact of the load on the skull was cut out, as well as in the left, undamaged hemisphere, in the same coordinates. Area—the parietal cortex of the brain. An excised piece of mouse cerebral cortex is fixed for 6 h in 4% paraformaldehyde and incubated for 48 h in 20% sucrose at 4 °C and then placed on a 4% agarose gel (Low-melting agarose, Sigma Aldrich, Burlington, MA, USA). Sections of agarose blocks with a thickness of approximately 20 μm are obtained on a Leica VT 1000 S vibratome (Germany). After washing with PBS, they are incubated for 1 h at room temperature with 5% BSA and 0.3% Triton X-100 to block non-specific binding sites. Sections are then incubated with rabbit primary anti-p53 antibody (1:100; PAA928Mu01, Cloud-Clone Corp, Wuhan, China) and mouse anti-neuN protein antibody (1:1000; FNab10266, FineTest, Wuhan, China) or astrocyte marker GFAP (1:1000; SAB4200571, Sigma Aldrich) for two days at 4 °C. After washing three times in PBS, sections were incubated with anti-rabbit IgG (H+L) Fluor488-conjugated (1:500; S0018, Affinity Biosciences, Zhenjiang, China) and anti-mouse IgG (H+L) Fluor594-conjugated (1:500) secondary antibodies (1:500; 500; S0005, Affinity Biosciences, Zhenjiang, China). Negative control-absence of primary antibodies. The nuclei of neurons and glial cells are visualized with Hoechst 33342 (40 μM; 10 min). Sections were placed in 60% glycerol and examined with an Altami LUM 1 fluorescence microscope (Ningbo Haishu Honyu Opto-Electro Co., Ltd., (Ningbo) China together with the company Altami, Russia) equipped with a high resolution digital camera (EXCCD01400KPA, Hangzhou ToupTek Photonics Co., Ltd., Hangzhou, China). Each group will include a minimum of 6 animals.

Colocalization of p53 with neuronal marker NeuN or GFAP was assessed using the Image J program with the JACoP plugin. The colocalization coefficient M1 reflects the proportion of pixels containing green (p53) and red signals (NeuN or GFAP) relative to the total signal recorded in the green channel [73]. At least 100 cells were used in the calculations. To quantify the average level of p53 fluorescence in experimental and control preparations, 10 control and 10 experimental images were used for each of the 6 mice. The average (by area) fluorescence of the cytoplasm and nucleus for each cell was estimated and the obtained values were averaged.

Six hours after axotomy, during the isolation of the cancer stretch receptor, an immunofluorescent study of the localization of the p53 protein in the CSR components was carried out. The primary antibodies were mouse anti-p53 antibodies (1:100; PAA928Mu01, Cloud-Clone Corp, China) diluted 1:100 in PBST-phosphate buffer (PBS: 10 mM phosphate buffer plus 2.7 mM KCl and 137 mM NaCl, pH 7.4, at 25 °C) containing 0.1% Tween 20. Anti-rabbit IgG (H+L) Fluor488-conjugated (1:500; S0018, Affinity Biosciences, China) diluted 1:500 in PBS. CSR preparations were fixed for 24 h with a 4% solution of paraformaldehyde in PBS and were then washed with a mixture of 1% bovine serum albumin (BSA), 0.2% NaN_3_ and 1% TritonX-100. Then, they were incubated for 24 h in a solution of primary antibodies, washed, incubated for 24 h in a solution of secondary antibodies, and washed again for 24 h at +4 °C.

To visualize the nuclei of glial cells and neurons, the preparations were fluorochromated with Hoechst-33342 (40 μM) for 10 min at +25 °C, then washed and placed in glycerol under a coverslip. CSR preparations were photographed using an Altami LUM 1 fluorescence microscope (Ningbo Haishu Honyu Opto-electro Co., Ltd., China together with the company Altami, Russia) equipped with a high-resolution digital camera (EXCCD01400KPA, Hangzhou ToupTek Photonics Co., Ltd., Hangzhou, China).

The level of p53 in various elements of the stretch receptor-the nucleolus, nucleus, and cytoplasm of the MRN, as well as in the glia surrounding the proximal segment of the axon, was estimated from the fluorescence intensity using the ImageJ program. The average (by area) fluorescence of the nucleus and cytoplasm of the neuron, the axon of the proximal and distal parts, and the nuclei of glial cells were evaluated. The measurement data were normalized to the background fluorescence intensity:

Inorm = (Imeas − Iback)/Iback,

where Imeas is the average intensity in the given area (nucleus, cytoplasm of MPH, axon, and glia) and Iback is the average background intensity.

### 4.4. Determination of Cell Death by the TUNEL Method

Determination of cell death of neurons and glial cells in TBI using the TUNEL method using the One-step TUNEL In Situ Apoptosis Kit (E-CK-A321, Green, AF488, Elabscience, Wuhan, China). This method allows the identification of apoptotic cells by terminal deoxynucleotidyl transferase (TdT)-mediated addition of labeled (X) deoxyuridine triphosphate nucleotides (X-dUTP) to the 3′-OH end of a DNA strand break, which can be detected using fluorescence microscopy.

Brain sections, 20 µm thick, were incubated with primary antibodies to the investigated NeuN and GFAP proteins, as described above. They were then glued onto glass slides coated with polylysine. The working solution for TUNEL marking was prepared immediately before use and kept on ice during its use. Sections were subjected to permobilization by inhibition in a working solution of Proteinase K (1:100) for 20 min at +37 °C. After the specified time, the preparations were washed 3 times for 5 min in PBS. Then, 100 µL of TdT balancing buffer was added to each preparation and incubated for 30 min at 37 °C. Then, the excess solution was carefully removed with filter paper, slightly dried, and 50 µL of the working solution was added to each section, incubated for 2 h in a humid chamber at 37 °C.

To evaluate the efficiency of determining apoptosis of neurons and glial cells on sections using the TUNEL method, a positive control was carried out by 10-min incubation of permeabilized sections with benzonase nuclease (1500 U/mL in 50 mM Tris-HCl buffer, pH7.5; 1 mg/mL BSA) at +25 °C. Sections were counterstained with Hoechst 33342, which visualizes the nuclei of neurons and glial cells (40 µM; 10 min).

The preparations were analyzed using a fluorescent microscope. TUNEL-positive cells were counted in 3 sections in the respective areas of the damaged and undamaged hemisphere, obtained from 6 animals for each group, which were injected with saline, Na_2_S and AOAA at 24 h and 7 days after TBI.

### 4.5. Determination of Cell Death in the Cancer Stretch Receptor

To visualize dead neurons and glial cells 6 h after axotomy, 20 μM propidium iodide and 10–20 μM Hoechst 33342 were added to the experimental bath. This time interval is necessary for the development of apoptosis. The slides were then washed several times with van Harreveld’s saline, fixed with 0.2% glutaraldehyde, washed several more times, and embedded in glycerol. Fluorescent images were studied using an Altami LUM 1 fluorescence microscope (Ningbo Haishu Honyu Opto-Electro Co., Ltd., Ningbo, China together with the company Altami, Russia) equipped with a high-resolution digital camera (EXCCD01400KPA, Hangzhou ToupTek Photonics Co., Ltd., Hangzhou, China). Propidium iodide gives red fluorescence to the nuclei of necrotic cells with a damaged plasma membrane, and Hoechst 33342 gives blue fluorescence to nuclear chromatin. It visualizes intact nuclei of living cells and fragmented nuclei of apoptotic cells. Nuclear fragmentation is the last stage of apoptosis when the point of no return has been passed. The percentage of red nuclei of necrotic glial cells and the number of fragmented nuclei of apoptotic glial cells were counted around the proximal segment of the axon 2 mm long, where glial cell apoptosis was more pronounced.

### 4.6. Immunoblotting

Expression of p53 under conditions of activation or inhibition of H_2_S-dependent signaling pathways in the penumbra region in the cerebral cortex of mice subjected to TBI and axotomized ganglia of VNC cancer was studied using the Western blot method.

To do this, 24 h or 7 days after TBI, the animals were decapitated, the brain was removed, and the infarction area was cut out and removed on ice with a 3 mm cylindrical knife, and then a ring with a 2 mm splint corresponding to the penumbra was cut out with another cylindrical knife with a diameter of 7 mm. The same rings (control samples) were excised from the contralateral cortex of the same mouse. After the samples were homogenized on ice with the addition of lysis buffer for 5 min (IS007 Lysis buffer-3, Cloud-Clone Corp, China), supplemented with a cocktail of protease and phosphatase inhibitors (PPC1010, Sigma-Aldrich), necessary for the preservation of proteins and their phosphorylated forms, as well as nuclease benzonase (E1014, Sigma-Aldrich), which is necessary for the destruction of nucleic acids. After homogenization, the samples were centrifuged for 20 min at 10,000–11,000× *g* at 4 °C in a Hanil Scientific M15R centrifuge equipped with a cooling system (Hanil Scientific Inc., Kimpo, Republic of Korea). Then, the supernatant was taken for analysis.

VNC samples after bilateral axotomy were incubated for 6 h in van Harreveld’s solution with or without H_2_S modulators at room temperature in the dark. For a sufficient amount of biomaterial, ganglia were combined from five experimental and control VNCs. Then, the sample preparation was carried out as described above.

Before electrophoresis, the protein content was determined using the Bradford reagent (K002-500, FineTest, China) on a SpectroSTAR Nano Plate Reader spectrophotometer (BMG Labtech, Ortenberg, Germany).

Samples containing 10–20 µg of protein per 15 µL were subjected to electrophoretic separation in a polyacrylamide gel (7–12%) in the presence of sodium dodecyl sulfate in a mini-PROTEAN Tetra cell (Bio-Rad, Hercules, CA, USA). Affinity Prestained Protein Ladder (KF8009, Affinity, China) was used as standard protein markers. After separation, the proteins were subjected to electrotransfer onto a PVDF membrane (polyvinyl difluoride membrane 162-0177, Bio-Rad) using the Trans-Blot^®^ Turbo Transfer System (Bio-Rad, USA). After washing with PBS, the membrane was successively incubated for 1 h in blocking buffer (TBS 1% Casein Blocker, Bio-Rad) and overnight at 4 °C with primary rabbit antibodies against p53 (1:500; PAA928Mu01, Cloud-Clone Corp, China) or Bax (SAB5700071, Sigma-Aldrich, St. Louis, MI, USA), Bcl-2 (SAB5700676, Sigma-Aldrich, United States) and β-actin (1:1000; PAB340Mi01, Cloud-Clone Corp, China). After incubation, membranes were washed in Tris buffer supplemented with 0.1% Tween-20 (TTVS, 10 mM; pH 8) and incubated for 1 h at room temperature with a secondary antibody against rabbit IgG peroxidase (1:1000; S0001, Affinity Biosciences, China).

Protein detection was performed using the KF8001 Affinity™ ECL kit for chemiluminescent detection of HRP conjugates (KF8001, Affinity Biosciences, China). Chemiluminescence was analyzed using the SH-Advance523 high resolution gel-documenting system (Shenhua Science Technology Co., Ltd. (SHST), Hangzhou, China). The resulting images were processed using the Vision Capt software package version 16.08.

### 4.7. Statistical Analysis

Statistical analysis was performed using one-way analysis of variance (ANOVA) with Dunnett’s post hoc test. The normality and homogeneity of the variance were assessed using the Shapiro–Wilk and Brown–Forsyth tests, respectively. If the normality or homogeneity of the variance was not confirmed, the non-parametric Kruskal–Wallis H test was used. All study results were analyzed blindly. The required animal sample size was calculated using the Piface software version 1.72. Power analysis demonstrated that a sample of 6 mice and 6 crayfish per group provided a detection power of 80% with a 5% probability of falsely rejecting the null hypothesis, which is an acceptable deviation. Differences were considered significant at *p* < 0.05 and n = 6. The data obtained are expressed as the mean ± standard error of the mean.

## 5. Conclusions

Thus, in the course of this study, it was shown that one of the mechanisms for regulating p53 expression in neurons and glial cells in TBI and axotomy can be H_2_S; in particular, H_2_S generated by the key enzyme of its synthesis in the nervous tissue CBS. The H_2_S donor, Na_2_S, showed a neuroprotective effect, manifested as a decrease in TUNEL-positive neurons and glial cells in TBI and apoptotic glia in axotomy. This effect could be realized through a Na_2_S-dependent decrease in the level of p53 in the cells of the nervous tissue of vertebrates and invertebrates. We observed the opposite effect when using the classic CBS inhibitor, AOAA. This indicates an important role of CBS in the survival and death of neurons and glial cells under conditions of cell stress responses to traumatic injury induced by TBI or axotomy. The accumulation of p53 in the proximal and especially distal parts of the MRN axon may indicate that p53 is directly involved in the processes developing in the damaged axon. Undoubtedly, our studies on three models of neurotrauma in vertebrates and invertebrates indicate H_2_S-dependent conservative mechanisms of regulation of p53 expression and cell death. The results obtained are of fundamental value on the role of H_2_S in the survival and death of neurons and glial cells. In addition, they can be used in the development of effective neuroprotective drugs of a new generation.

## Data Availability

Not applicable.
